# Comparative SNP diversity among four *Eucalyptus *species for genes from secondary metabolite biosynthetic pathways

**DOI:** 10.1186/1471-2164-10-452

**Published:** 2009-09-24

**Authors:** Carsten Külheim, Suat Hui Yeoh, Jens Maintz, William J Foley, Gavin F Moran

**Affiliations:** 1 Research School of Biology, Australian National University, 116 Daley Road, Canberra, Australia; 2 Department of Botany, Ruhr Universität, Bochum, German

## Abstract

**Background:**

There is little information about the DNA sequence variation within and between closely related plant species. The combination of re-sequencing technologies, large-scale DNA pools and availability of reference gene sequences allowed the extensive characterisation of single nucleotide polymorphisms (SNPs) in genes of four biosynthetic pathways leading to the formation of ecologically relevant secondary metabolites in *Eucalyptus*. With this approach the occurrence and patterns of SNP variation for a set of genes can be compared across different species from the same genus.

**Results:**

In a single GS-FLX run, we sequenced over 103 Mbp and assembled them to approximately 50 kbp of reference sequences. An average sequencing depth of 315 reads per nucleotide site was achieved for all four eucalypt species, *Eucalyptus globulus*, *E. nitens*, *E. camaldulensis *and *E. loxophleba*. We sequenced 23 genes from 1,764 individuals and discovered 8,631 SNPs across the species, with about 1.5 times as many SNPs per kbp in the introns compared to exons. The exons of the two closely related species (*E. globulus *and *E. nitens*) had similar numbers of SNPs at synonymous and non-synonymous sites. These species also had similar levels of SNP diversity, whereas *E. camaldulensis *and *E. loxophleba *had much higher SNP diversity. Neither the pathway nor the position in the pathway influenced gene diversity. The four species share between 20 and 43% of the SNPs in these genes.

**Conclusion:**

By using conservative statistical detection methods, we were confident about the validity of each SNP. With numerous individuals sampled over the geographical range of each species, we discovered one SNP in every 33 bp for *E. nitens *and one in every 31 bp in *E. globulus*. In contrast, the more distantly related species contained more SNPs: one in every 16 bp for *E. camaldulensis *and one in 17 bp for *E. loxophleba*, which is, to the best of our knowledge, the highest frequency of SNPs described in woody plant species.

## Background

The genome sequence is known for a limited number of plant species including *Arabidopsis thaliana, Oryza sativa, Vitis vinifera *and *Populus trichocarpa *[1-5]. In a few plant species, levels of polymorphism in DNA sequences have been estimated using large genome data sets [6-8]. Further, in a study of *A. thaliana*, 20 accession lines were studied to determine SNP variation at genome-wide level [9]. There are two limitations to estimating complete DNA sequence variation in a species. First, it requires comprehensive sampling of individuals, which is costly and time consuming. Second, it demands the most modern technology. Next generation sequencing (NGS) provides opportunities for cost-effective sequencing of massive amounts of DNA. The relatively new method of parallel pyrosequencing developed by 454 Life Sciences has quickly improved in the number of reads and read length. Two studies recently used these technologies to test fidelity of sequence variants from pooled DNA comprising DNA from several individuals [10,11]. Both studies proved that with adequate depth of sequence coverage it was possible to detect and estimate the frequencies of SNPs. Such an approach has been used to identify many SNPs in domestic pigs (*Sus domestica *Linn.) [12], rhesus macaques (*Macaca mulatta*, Zimmermann) [13], whole genome comparisons of bacteria (Salmonella (*Salmonella spp*.) [14], and from seven families of *Eucalyptus grandis*, Hill [15].

Missing from plant biology are large comparative studies of genomes from closely related plant species [16]. Soon it will be easy to sequence and thus compare closely related genomes, for example from relatives of *Arabidopsis *or rice. In other sets of closely related species, for which there is no genome sequence, total sequence diversity at a set of orthologous genes can be compared. Simultaneously, with the use of genes in defined biosynthetic pathways, associations between levels of SNP variation and the position of the genes in the pathway can be examined and also comparisons made of the levels of gene diversity between pathways. For instance, in *A. thaliana *there was no association between variation and position in the pathway for a set of genes from the phenylpropanoid pathway [7]. In eucalypts, we are interested in the terpenoid and flavonoid pathways, because they produce compounds that are important in the plants' response to stress and herbivory. Furthermore, these pathways are well defined in several plant species [17-21], but there have been no genetic studies of the genes in these pathways in eucalypts.

The four *Eucalyptus *species used in this study all belong to the subgenus *Symphyomyrtus*. The two major commercial species, *E. globulus *and *E*. nitens, are closely related and belong to the same series (*Globulares*) and section (*Maidenaria*). The other two, *E. camaldulensis *and *E. loxophleba*, are in different sections, *Exsertaria *and *Bisectae*, respectively [22]. There is uncertainty about the dates of divergence of lineages within the subgenus *Symphyomyrtus *but *E. globulus *and *E. nitens *probably diverged 5 - 10 million years ago, while the more distantly related *E. loxophleba *might have diverged between 20 - 40 million years ago [23,24]. All are important species for wood and pulp production or are planted widely to ameliorate salinity.

We sequenced 23 genes from the gDNA of 1,764 individuals of four different *Eucalyptus *species allowing us to characterise the SNPs in these genes and to compare their patterns of occurrence among species.

## Results

### Pooled samples from individuals collected across the geographic range of four Eucalyptus species

For comprehensive characterisation of SNP variation within an outcrossing plant species, sampling must include a large number of individuals from populations covering the geographic range. The samples used in this study cover the complete natural geographic range of four *Eucalyptus *species (Table 1). *E. globulus *and *E. nitens *are represented by 46 and 28 populations, respectively whereas for *E. camaldulensis *and *E. loxophleba *a total of 93 and 29 populations were sampled [22,25,26]. With the combination of large numbers of populations and individuals for each species, we were confident of discovering all common alleles and the majority of rare alleles for the genes of interest. Since a bulk sample of pooled individuals was used for each species, it was important that the sequences sampled from the pool were a random selection of those from all individuals. Most of the 12 individuals from each of the four species tested for the 57 primer pairs gave single PCR products of correct size with no apparent bias in success rate over the geographic range (data not shown). For the 96 individuals *of E. globulus*, an average of 94 (98%) gave a single PCR product from each of the 17 amplicons tested (data not shown). These data suggest that the primers work equally well for all samples.

**Table 1:** *Eucalyptus *species included in this study with number of populations and number of individuals sampled for each species.

**Species**	**Subspecies**	**Populations**	**Individuals**	**Geographic distribution^a^**
*E. globulus*		46	511	Tas, S-Vic
*E. nitens*		28	381	E-Vic, SE-NSW
*E. camaldulensis*	6	93	456	WA, NT, QLD, SA, NSW, VIC
*E. loxophleba*	4	29	416	S-WA

	**10**	**196**	**1764**	

^a ^Tas: Tasmania; S-Vic: South Victoria; E-Vic: East Victoria; SE-NSW: South-east New South Wales; WA: Western Australia; NT: Northern Territory; QLD: Queensland; SA: South Australia; NSW: New South Wales; VIC: Victoria; S-WA: South Western Australia

### Genes of the terpenoid and flavonoid pathway

We used EST sequences from public libraries to design specific primers for 37 genes. The primers were used to amplify *E. globulus *gDNA and the genes were sequenced with the traditional Sanger method. These sequences served as the reference sequences for later assembling the sequences, which were obtained from the 454 pyrosequencing data. Table 2 shows the 23 genes for which SNPs were obtained in our re-sequencing study. When multiple genes of a gene family were included the genes are numbered sequentially (1, 2, etc.). Nine of the Eucalyptus gene sequences covered the entire corresponding *Arabidopsis *gene. The structure of the reference sequences, in terms of the number of exons and introns, was similar to that in model plant species. The exons of the eucalypt genes were similar in size to those in *Arabidopsis*, but the size of the eucalypt introns varied widely from *Arabidopsis*. The introns were mostly larger in eucalypts compared to *Arabidopsis*. At the translated amino acid level the reference sequences of *E. globulus *were on average 75%identical to those in *Arabidopsis *(Table 2).

**Table 2:** Details of 23 genes of secondary metabolisms in *Eucalyptus *sequenced for SNP detection

		**Length (bp)^a^**					
					
**Gene**	**aa identity (%)^b^**	**Exon**	**Intron**	**Total**	**Pathway**	**Coverage (%)**	**Coverage**
*dxr*	75	652	1440	2092	MEP	80	E1-E6
*dxs1*	83	1158	668	1826	MEP	57	I4-E10
*dxs2*	82	588	184	772	MEP	30	E5-E7
*hds*	75	2001	2997	4998	MEP	93	E1-E17
*hdr*	83	1218	1982	3200	MEP	100	E1-E10
*hmgr*	73	890	1573	2463	MVA	75	E1-E2/E6-E12
*mvk*	62	1016	2023	3039	MVA	100	E1-E5
*pmd*	76	484	1472	1956	MVA	59	E1-E4
*ipp*	83	598	1138	1736	TPS	76	E2-E6
*ggpps*	73	731	448	1179	TPS	90	E1-E2
*psy1*	80	742	819	1561	TPS	80	E1-E4
*psy2*	75	617	882	1499	TPS	80	E1-E4
*psy3*	85	423	733	1156	TPS	40	E4-E6
*gpps*	63	568	4123	4691	TPS	75	E3-E8/E9-E10
*fpps*	73	910	2563	3473	TPS	100	E1-E12
*smo*	78	1486	861	2347	TPS	100	E1-E7
*chs*	66	1126	362	1488	FLAV	100	E1-E2
*chi*	70	808	357	1165	FLAV	100	E1-E3
*f3h*	72	1055	963	2018	FLAV	100	E1-E3
*dfr*	72	921	1439	2360	FLAV	100	E1-E6
*lar*^c^	65	569	2097	2666	FLAV	80	E1-E4
*ans*	71	952	216	1168	FLAV	100	E1-E2
*anr*	68	534	545	1079	FLAV	63	E1-E4

sum	75	20047	29885	49932		81	

^a ^The length of sequence for exons, introns and the total are shown, the assigned pathway, an estimation of the proportion of the full length of the gene that was sequenced, the coverage of the gene, as compared to *A. thaliana*.

^b ^Compared to *A. thaliana*

^c ^*lar *is compared to *P. trichocarpa *as it does not exist in *A. thaliana*

### Pyrosequencing and reference assembly

The separate barcodes for each of the four *Eucalyptus *species successfully enabled separation of the DNA sequences by species after the completion of the 454 sequencing. The data then consisted of 473,182 sequences with an average length of 219 bp, giving 103.6 Mbp of sequence (Table 3). Of these, we could align 81% to our reference sequences, while only 1% of the sequences represented contamination (chloroplastic-, mitochondrial-, and human- DNA). Three genes were discarded because they had too few reads aligned to the reference sequences. The remaining 18% of the sequences could be assembled into contigs belonging to members of large gene families, such as the terpene-, chalcone- and squalene- synthase families. Some genes within these families are highly conserved in the coding regions and can be distinguished only in the more diverse introns. Primers were required that would amplify genes from different species and populations, so we selected conserved regions in the exons for primer design. However, as a result, some primer pairs amplified genes from multiple loci. These genes were not used for our SNP analysis since our focus was the discovery of discrete single nucleotide polymorphisms (SNPs) for association studies. Hence the number of genes analysed was reduced from 37 to 23. The average read length was the same across species. The number of reads and total length of sequence varied substantially among species with the figures for *E. globulus *about two-thirds of those for *E. loxophleba *(Table 3), which may have been due to unequal amounts of DNA used in the barcoding or pyrosequencing process. These estimates do not appear to relate to the detection rates of SNPs (Table 4).

**Table 3:** Summary statistics of the re-sequencing experiment of four species of *Eucalyptus*.

	***E. globulus***	***E. nitens***	***E. camaldulensis***	***E. loxophleba***	**SUM/average**
Total No. reads	99,452	114,423	113,063	146,244	473,182
Matched to reference	75,558	94,619	93,200	121,377	384,754
Matched (%)	76	83	82	83	81
Average read length (bp)	217	217	220	221	219
Total sequenced (bp)	21,581,084	24,829,791	24,873,860	32,319,924	103,604,659

**Table 4:** The absolute number of SNPs in the exons and introns of four species of *Eucalyptus*.

	**Allele type**^a^	***E. globulus***	***E. nitens***	***E. camaldulensis***	***E. loxophleba***	**sum**
Exons	common synonymous	96	63	142	136	437
	common non-synonymous	82	30	114	97	323
	rare synonymous	133	175	358	316	982
	rare non-synonymous	174	203	273	238	888
	
	**total**	**485**	**471**	**887**	**787**	**2630**

Introns	common	367	344	634	720	2065
	rare	626	603	1510	1197	3936
	
	**total**	**993**	**947**	**2144**	**1917**	**6001**

Exons + Introns	common	545	437	890	953	2825
	rare	933	981	2141	1751	5806
	
	**total**	**1478**	**1418**	**3031**	**2704**	**8631**

The number of SNPs are shown as the number of SNPs detected. ^a ^Common allele is ≥ 10%, rare allele is <10%

### SNP detection and analysis

A total of 8,631 SNPs were detected in the four eucalypt species. Of these, 2,825 were common and 5,806 were rare SNPs (Table 4). The use of pooled DNA precluded the derivation of haplotypes of individuals and meant that we could not make nucleotide diversity estimates. Two species, *E. camaldulensis *and *E. loxophleba*, had approximately twice as many SNPs as the other two species (Table 4). Common SNPs that lead to changes in the amino acid (exons, common non-synonymous) were fewest, while rare SNPs in introns were most frequent. The proportion of common SNPs that were non-synonymous ranged from 32% in *E. nitens *to 46% in *E. globulus*. The proportion of SNP sites in the exons that are non-synonymous is high (ca 50%) in both *E. globulus *and *E. nitens *but slightly less (ca 43%) in the other two species. Table 5 shows the number of SNPs per 1,000 bp of data, categorised according to rate of occurrence, synonymity and position (exons or introns), allowing a better comparison of the SNP frequencies among species. *Eucalyptus nitens *has the lowest frequency of common SNPs in the exons and of all SNPs in the introns, while *E. globulus *has the lowest frequency of rare SNPs in the exons (Table 5). The other species share the highest SNP frequencies, with *E. loxophleba *being highest in two categories (exons, common synonymous and introns, common) and *E. camaldulensis *being highest in four. The ratio of SNPs in the introns to those in the exons was higher in *E. camaldulensis *and *E. loxophleba *than it was in *E. globulus *and *E. nitens *(1.70 and 1.69 *vs*. 1.41 and 1.39), indicating that the former have relatively more SNPs in the introns. At the species level, there was between one SNP in every 33 bp in *E. nitens *and one SNP in every 16 bp in *E. camaldulensis*.

**Table 5:** The normalized number of SNPs in the exons and introns of four species of *Eucalyptus*.

	**Allele type**	***E. globulus***	***E. nitens***	***E. camaldulensis***	***E. loxophleba***	
Exons	common synonymous	5.3	3.3	7.3	7.4	
	common non-synonymous	4.3	1.6	5.9	5.2	
	rare synonymous	7.6	9.5	19.0	17.4	
	rare non-synonymous	9.9	11.1	14.5	13.1	
	
	total	27.1	25.5	46.7	43.1	

Introns	common	13.7	12.2	22.7	26.7	
	rare	24.6	23.3	56.6	46.3	
	
	total	38.3	35.5	79.3	73.0	

The number of SNPs normalized to 1,000 bp

Table 6 shows the number of SNPs per 1,000 bp, for the introns and exons of individual genes. Generally, genes in *E. nitens *and *E. globulus *have similar levels of SNP diversity, especially in the exons, but the levels are much higher in *E. camaldulensis *and *E. loxophleba*. The gene with the lowest SNP frequency (across all species) was anthocyanidin synthase (*ans*) followed by geranylgeranyl diphosphate synthase (*ggpps*) and dihydroflavonol 4-reductase (*dfr*). In contrast, the gene with the highest SNP frequency was mevalonate kinase (*mvk*), followed by chalcone synthase (*chs*) and mevalonate diphospate decarboxylase (*pmd*). The SNP diversity in the exons of *mvk *is at least twice that of the exons in *pmd*.

**Table 6:** The frequency of SNPs per 1,000 bp for 23 genes of four biosynthetic pathways in each of four *Eucalyptus *species.

		**Exons**				**Introns**			
			
**Pathway**	**Gene**	**G^a^**	**N**	**C**	**L**	**G**	**N**	**C**	**L**
MEP	*dxr*	21.8	23.6	39.1	44.5	56.6	41.8	52.9	47.5
	*dxs1*	18.7	18.7	42.5	28.9	48.9	43.5	70.7	70.7
	*dxs2*		16.9	21.5	27.6		25.0	83.3	95.1
	*hds*	57.0	40.0	60.5	32.1	64.4	40.4	99.4	65.1
	*hdr*	5.7	20.5	47.6	36.9	19.7	40.8	83.5	111.0

MVA	*hmgr*	16.9	24.7	42.7	33.7	36.6	20.6	86.8	44.8
	*mvk*	40.4	38.4	52.2	61.0	56.9	82.0	67.4	112.2
	*pmd*	20.7	20.7	16.5	20.7	57.5	70.5	108.3	112.9

TPS	*ipp*	16.7	20.1	35.1	30.1	30.8	41.3	80.0	40.4
	*ggpps*	9.6	10.9	27.4	19.2	26.8	33.5	62.5	40.2
	*psy1*	21.2	26.9	90.7	32.6	38.4	46.2	91.9	53.0
	*psy2*	14.6	17.8	47.0	34.0	33.5	42.5	114.7	79.7
	*psy3*	21.3	21.3	30.7	28.4	28.6	23.2	54.6	51.8
	*gpps*	12.1	14.1	20.2	12.1	38.4	27.3	104.7	63.7
	*fpps*	11.2	8.7	17.4	43.5	26.0	25.8	52.5	79.1
	*smo*	27.2	21.3	35.3	54.5	36.8	29.4	44.1	51.5

FLAV	*chs*	20.4	18.7	37.3	63.1	76.3	61.3	122.9	88.4
	*chi*	3.4	8.1	16.4	32.7	50.5	24.8	54.5	67.6
	*f3h*	18.0	11.4	25.6	46.4	43.6	40.5	58.2	118.4
	*dfr*	13.0	7.6	33.7	19.5	22.8	24.7	55.5	63.0
	*lar*	17.9	18.9	49.4	34.7	41.7	37.0	83.3	88.0
	*ans*	7.0	7.0	35.1	24.6	18.7	11.6	50.4	36.8
	*anr*	11.2	91.8	31.8	26.2	20.2	86.2	60.6	56.9

^a^G = *E. globulus*, N = *E. nitens*, C = *E. camaldulensis*, L = *E. Loxophleba*

### SNP frequencies across the different pathways

**Figure 1:**
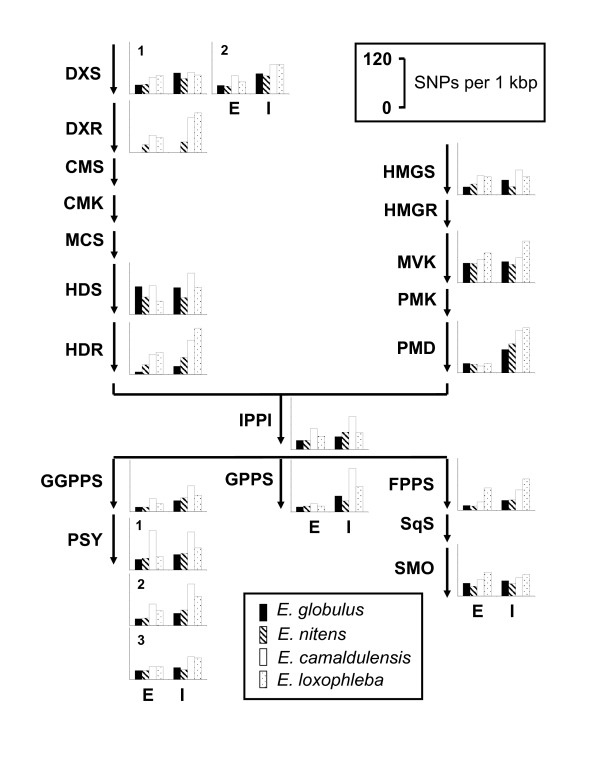
**Terpenoid biosynthetic pathways including SNP frequency**. Schematic showing the assumed biosynthetic pathways for terpenoids in eucalypts. For each gene with data present, the number of SNPs per 1,000 bp for all four species is shown. Scales and species depicted on the side. Exons (E) and introns (I) are shown separately.

**Figure 2:**
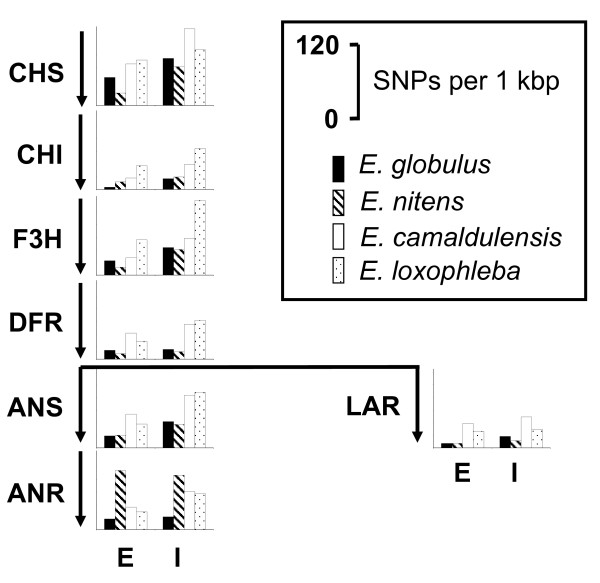
**Flavonoid biosynthetic pathway including SNP frequency**. Schematic showing the assumed biosynthetic pathway for flavonoids in eucalypts. For each gene with data present, the number of SNPs per 1,000 bp of each species is shown. Exons (E) and introns (I) are shown separately.

Genes at a bottleneck position of a pathway may be under stronger selective pressure and may therefore have fewer SNPs than will genes at other positions. Furthermore, the number of genes with the same function may determine the SNP frequency at a particular gene. We applied a two-tailed Students t-test to determine whether pathway position or gene copy number effect SNP frequencies. Figures 1 and 2 show the assumed terpenoid and flavonoid biosynthesis pathways in eucalypts together with the frequency of SNPs in the exons and introns within each gene for all four species (Table 6). It is apparent that for most genes the two *Eucalyptus *species, *E. camaldulensis *and *E. loxophleba *have more SNPs per kbp than do the other two species. This is true for both exons and introns. Further analyses showed that the introns of the mevalonate pathway (MVA) have significantly more SNPs than do those of the terpenoid pathway (TPS) (*P *= 0.02), no other combination was significantly different when taking averages of all four species. Within species, however, we found two significant differences, both in *E. globulus*. The MVA pathway had significantly more SNPs in the exons than did the flavonoid pathway (FLAV) (*P *= 0.05), while the MVA pathway had more SNPs in the introns than did the TPS pathway (*P *= 0.01).

We hypothesized that genes that occur only once within the genome (single genes) are under higher selective pressure and therefore we expected to observe less SNPs in those genes than genes that occur in multi-gene families. Additional file 1 shows how many members there are in each gene family from some species with known genome sequences (*Arabidopsis thaliana*, *Oryza sativa, Populus trichocarpa *and *Vitis vinifera*). BLAST searches within the genome web pages (*A. thaliana *(TAIR v. 7.0), *O. sativa *(TIGR v. 5.0) and *P. trichocarpa *(JGI v. 1.1)) and searches for the gene name in well-annotated genome web pages (*A. thaliana *and *O. sativa*) generated most of the data, while the numbers for *V. vinifera *came from Velasco and co-workers [5]. A comparison of our results to those of Tsai and co-workers [27], who used similar methods to estimate the gene numbers for flavonoid biosynthesis pathway genes from *A. thaliana *and *P. trichocarpa*, indicated few differences, which were of little consequence in placing the genes into two categories: single or low copy and high copy number genes (see Additional file 1). The occurrence of pseudogenes make it difficult to verify the exact copy number of a gene and, in the case of *Eucalyptus*, the imminent release of the genome sequence will make accurate measures easier. A comparison of SNP frequencies between single or low copy genes and genes from large families showed no significant differences. There appears to be a tendency towards higher SNP frequencies for genes in the MVA pathway than for genes in other pathways (Table 6), but definite conclusions require data on the *hmgr *and *pmk *genes

### Comparing SNPs among Eucalyptus species

The four species studied have high sequence homologies for the genes analysed, with often less than three nucleotides difference per 1,000 bp in exons separating them. The number of SNPs that each species shared with at least one other species is shown in Table 7. The estimates are very similar for *E. globulus *and *E. nitens*, both for the exons and the introns (Table 7). In contrast, *E. camaldulensis *and *E. loxophleba *share 274 and 204 SNPs from the exons and 519 and 399 SNPs from the introns. While the absolute number of shared SNPs is higher for the latter two species, the proportion is lower. Overall the proportion of SNPs shared by more than one species is high - 33%.

**Table 7:** The number and percentage of SNPs in either the exons or introns shared by more than one species of *Eucalyptus*.

	***E. globulus***	***E. nitens***	***E. camaldulensis***	***E. loxophleba***
Exons	192 (39.6)^a^	187 (39.7)	274 (30.9)	204 (25.9)
Introns	430 (43.3)	378 (39.9)	519 (24.2)	399 (20.8)

^a ^The percentage of the total number of SNPs

### Analysis of synonymous and non-synonymous sites across Eucalyptus

Genes can be under positive or negative selection. Insight into the type of selection can be obtained from the ratio of non-synonymous mutations per non-synonymous site to synonymous SNPs per synonymous site in the exons of a gene (pN/pS). Ratios for genes that had less than ten variants were excluded, for example *E. globulus lar *and *anr *with four and nine variations, respectively. We could not determine a value for *E. globulus *chalcone isomerase (*chi*), because it lacked a synonymous mutation. The values of pN/pS ranged from 0.04 to 0.95, averaging 0.3. The average ratios for the four species were between 0.08 and 0.69 indicating purifying selection in the pathways studied (see additional file 2). Interestingly, the two genes with the lowest pN/pS ratio were 1-deoxyxylulose-5-phosphate synthase 1 (*dxs1*) and 1-deoxyxylulose-5-phosphate synthase 2 (*dxs2*).

## Discussion

For the 23 genes 8,631 high confidence SNPs across the four species were identified from 1,764 individuals that represented the full geographic range of each species. Our strict criteria undoubtedly led to the exclusion of many 'real' SNPs, but ensured that there were no false-positives in our SNP identification. There should be no ascertainment bias due to our sampling approach, however, a major potential bias has been raised with re-sequencing methods, namely PCR amplification from pooled samples [11,28]. Pools of DNA from four species were used in this SNP discovery project and the primers were designed inside the exons to enhance our chance of even and equal amplification within and across all pools. Our success rate for using primers across all species was 100%. Nevertheless, this came at a cost, since we had to exclude data from genes where the primers clearly amplified more than one locus, namely the large gene families of terpene-, squalene- and chalcone- synthases. The study was successful because the reference or consensus sequences in the four species showed high identity. It is unclear whether exclusion of gene families led to bias in estimation of overall levels of SNP diversity.

A general conclusion that can be drawn for the four species is that within all genes there is more SNP variability in introns than in the exons. In only three of the 92 comparisons (*hmgs *and *anr *in *E. nitens *and *smo *in *E. loxophleba*) do more SNPs occur in exons than introns. The lowest SNP density in the exons was found in *E. nitens *with one SNP in every 39 bp, and the highest in *E. camaldulensis *with one SNP in every 21 bp, which is similar to estimates of one in 43 bp and one in 50 for ESTs from maize [29,30]. For angiosperm trees comparable estimates are one in 25 bp for *Quercus crispula *[31] and one every 60 bp in *Populus tremula *[32]. In all these studies a limited number of individuals were sampled. Our estimates are much higher than comparable SNP frequencies previously reported in *Eucalyptus *of one SNP in every 192 bp [15]. This could be explained by our experimental design, which examined a comprehensive set of populations over the geographical range of each species, in contrast to *E. grandis *where only three individuals from each of seven families were examined [15].

There are different levels of SNPs between genes within species. *Eucalyptus camaldulensis *and *E. loxophleba *have higher levels of polymorphism for individual genes than *E. globulus *and *E. nitens*, but there is a smaller range in SNP polymorphisms between genes. A high proportion of the discovered SNPs are shared between several species. While some of these may have occurred independently after species separation, many would have been present before the speciation events. If evolutionary time from speciation is the dominant factor then one would expect that genes from other biosynthetic pathways in the same eucalypt species will show similar patterns. There is much uncertainty as to how long ago the species separated. The proposed separation age ranges from 5 - 10 mya for *E. globulus *and *E. nitens *to 20 - 42 mya for *E. loxophleba *from the other three species [23,24]. That so many SNPs are in common between species suggest selective forces have maintained many of them over this period. Similarly, the unusually large proportion of non-synonymous SNP sites, especially of common SNPs, along with the high similarity of proportions of synonymous versus non-synonymous SNPs across species suggests maintenance of these SNPs through selection.

It is noticeable that the two sister species *E. globulus *and *E. nitens *have very similar levels of SNP diversity overall and at the intron and exon level. The similar proportions of common SNPs in introns for *E. globulus *and *E. nitens *could result from evolutionary lineages of comparable age. In fact, they share about 28% of their SNPs, even though they have been separated for several million years. Morphologically they are quite distinct species. For the ten other species in the small taxonomic group *Globulares *to which these two species belong [22], we hypothesise that similar patterns of polymorphism will be found for the same functional set of genes. Will other eucalypt species pairs show similar patterns and what does it infer about evolutionary relationships within and between groups of species?

*Eucalyptus camaldulensis *has the highest numbers of SNPs, especially of rare alleles both in the exons and introns. This species has the largest geographic range of any eucalypt species and the most number of natural populations of the four species that were sampled. Perhaps the species with the greater number of separate evolving populations will have a greater array of rare SNPs. A SNP data set from individuals rather than pooled bulks of DNA would allow examination of this hypothesis. The several subspecies within both *E. loxophleba *and *E. camaldulensis *suggest greater evolutionary divergence within these species and this seems to be reflected in the higher SNP diversity in the two species. The higher intron/exon ratios of SNPs in these two species could reflect that they represent older evolutionary lineages which have enabled greater accumulation of SNP alleles over time, especially in introns, where selective forces could be weaker. Similar differences in intron/exon SNP ratios may occur in other eucalypts and plant groups as a result of differences in length of evolutionary lineages.

In a study of nine genes of the phenylpropanoid pathway in *A. thaliana *no association was detected between sequence diversity and position in the pathway [7]. In our study structural genes of the terpenoid and flavonoid biosynthesis pathways, which are important in plant-herbivore interactions [33-35], were used. Essentially, no relationship was found between the levels of SNP diversity in genes and their position in the pathways or between pathways. Even without some gene sequences the coverage of the pathways was sufficient to make these conclusions. Most of the genes studied appear to be under purifying selection. Similar results have been reported in other plants [15,36]. In forest trees current data suggest 15-20% of genes are under some form of selection [37,38]. It is possible that the assumption for a genome-wide neutral model does not apply for the eucalypt species. Whether many of the observed patterns are due to common demographic factors rather than selection may be resolved when nucleotide diversity estimates are available at the population level.

The hypothesis that entry point enzymes such as *dxs *control the downstream production of terpenoids [39] is not reflected in lower levels of SNP diversity in the corresponding genes, but may be reflected by the low ratios of pN/pS. Nevertheless there could be significant associations between SNP polymorphisms and concentrations of final products in these genes. Furthermore, we only examined structural genes here and there could be strong selection on the unknown regulatory elements involved in the pathway. Recent studies have found evidence of different patterns of polymorphism between different functional gene classes with genes interacting with the environment having high levels of SNP diversity [9]. Examination of the data set in this study with genes in pathways responsible for other phenotypic traits for the same eucalypt species and individuals will enable similar comparison

## Conclusion

Our study shows that it is possible to discover most common SNPs and a large proportion of rare sequence variants by 454 pyrosequencing for fragments amplified from species-wide pooled DNA in a set of targeted genes. We emphasize that a comprehensive sample collection is the key for comprehensive SNP discovery. With one SNP in every 16 bp *E. camaldulensis *has the highest SNP frequency of any woody plant species studied so far. The high number of shared SNPs between *E. globulus *and *E. nitens*, as well as the similar patterns of SNPs in all studied genes is a reflection of their close phylogenetic relationship. The study successfully characterised a large number of common SNPs that can be used in association studies in eucalypts.

## Methods

### Plant collection

We collected *Eucalyptus globulus *(Labill.) at a field trial in Northern Tasmania (Latrobe site). This trial was planted in 1989 from open pollinated seeds collected from 46 locations throughout the geographic range of the species [40-42]. Leaves from 511 individual trees collected in September 2006, were frozen immediately and kept at -80°C until further use. Leaves and DNA from 381 *Eucalyptus nitens *(Maiden) trees were previously obtained as part of association genetic studies of the central highlands regions of Victoria [43] and also from a population genetics study of *E. nitens *[44]. The four NSW populations (83 trees) from the Byrne study [44] along with 24 central Victorian populations completed the geographic coverage for *E. nitens *(Table 1). Butcher had previously collected leaf samples and extracted DNA from populations of *E. camaldulensis *(Dehnh.) covering the geographic range [26,45]. We used DNA samples from 456 of these sample trees representing 93 populations. Finally we collected leaves from 416 *E. loxophleba *(Benth.) from 29 populations in 2007/2008 covering the range of the species, including the four subspecies [46].

### DNA extraction

Genomic DNA (gDNA) was isolated from *E. globulus, E. nitens *and *E. camaldulensis *with a modified CTAB method [47]. and from *E. loxophleba *with a Qiagen DNeasy 96 Plant kit (Qiagen Australia, Doncaster, Vic, Australia). We measured the gDNA concentration of each individual sample with a NanoDrop ND-1000 spectrophotometer (Thermo Scientific, Wilmington, DE, USA) and adjusted the concentration to 50 ng/μl for *E. globulus, E. nitens *and *E. camaldulensis *and to 25 ng/μl for *E. loxophleba*. Finally, we pooled DNA samples from each species and checked the resulting pool for quality and quantity on a ND-1000 (Thermo Scientific, USA).

### Gene discovery and primer design

Amino acid sequences from genes of the terpenoid and flavonoid biosynthetic pathways were obtained from *Arabidopsis thaliana *and in some cases *Populus trichocarpa*. They were then used to search for eucalypt sequences with a BLAST search against the Genebank  EST data base (tblastn against Myrtaceae). *Eucalyptus *hits were then aligned for each gene, translated into protein and reverse BLAST was used against Genebank to confirm the gene identity. Primers were designed (see Additional file 3) to amplify each gene from gDNA to gain intron sequences as well as confirming the exon sequences. Some sequences (*chs *and *tps*) came from degenerate primers, cloning, and sequencing in our laboratory.

Many of the ESTs did not cover the complete open reading frame of its gene so to close gaps on the 5' end of these genes we used the GenomeWalker Universal Kit (Clontech Laboratories, Mountain View, CA, USA). From the combined sequence information we made a consensus sequences for each gene, which later served as our reference sequence for the 454 pyrosequencing assembly. We designed primers for 37 genes starting within the first available exon and ending inside an exon, to produce a fragment of maximum 2,100 bp. If required, multiple primer pairs were designed to fully cover a gene (see additional file 3), giving us 57 amplicons. The combined length of all amplicons was approximately 89 kbp.

### Verification of primers and fragment amplification

We verified that all primers worked within a species using firstly 12 individuals from each species which were selected to cover their geographic ranges, and secondly for 96 individuals from *E. globulus *but for a subset of 17 amplicons. Fragments were amplified by PCR with TAQ-Ti (Fisher-Biotech, West Perth, Australia) using standard PCR conditions, separated on 1.2% Agarose gels containing ethidium bromide with images captured on a Molecular Imager Gel Doc XR System (Bio-Rad Laboratories, Hercules, CA, USA). For re-sequencing, we amplified fragments from each of the four pools of gDNA by PCR under the conditions described previously and then ran a fraction of the PCR product on an agarose gel to check its quality. We cleaned single fragments of the expected size with a QIAquick PCR purification kit (Qiagen, Doncaster, Vic, Australia). When there were several fragments, we separated the PCR products on a 1.2% Agarose gel and extracted them from gel using a Qiaquick Gel Extraction Kit (Qiagen, Australia). The DNA concentration of each purified fragment was then quantified on a ND-1000 (Thermo Scientific, USA) and equimolar amounts of all fragments from each species were pooled. Each pool was checked for quality and quantity by assay on a ND-1000 (Thermo Scientific, USA) and by Agarose gel electrophoresis. The fragment pools were frozen and shipped cold to the Australian Genome Research Facility (AGRF, St Lucia, QLD, Australia).

### 454 sequencing and assembly

After nebulisation, we barcoded the pool from each species and had it sequenced on a Life Sciences GS-FLX according to standard procedures (454 Life Sciences, Branford, CT, USA). Sequences were "base-called" using 454 software and then separated according to the barcode. The sequences that did not have a functional barcode were discarded (<0.7%). We truncated those sequences with low base-call quality before separately assembling the sequences of each species to the reference gene sequences using CLC Genomics Workbench software (CLC bio, Aarhus, Denmark) with the software's standard assembly parameters. For each gene, we excluded regions of low read depth (<20). For unknown reasons there were no sequences for the *E. globulus dxr *gene. Files containing reads' sequences and quality scores were deposited in the Short Read Archive of the National Center for Biotechnology Information (NCBI) [accession number SRA008618].

### SNP detection and analysis

We used the CLC Genomics Workbench software (CLC bio, Denmark) to detect single nucleotide variants within each species. After reference assembly we had average sequencing depths between 242 and 410 reads at each nucleotide site (average of 315). We used a SNP discovery window of 7 bp at a central base quality score of 40 with surrounding base quality scores of 20 or more. At read depths between 20 and 50, we kept only alleles at a frequency of at least 10%, whereas at read depths over 50, any allele frequencies were kept as long as there were at least 3 reads with the allele variant. Our confidence criteria lead to the exclusion of 3,734 SNPs. We designated alleles "rare" if they occurred at frequencies below 10% and "common" when their frequencies equalled or exceeded 10%. Finally, we counted the number of SNPs for each gene and normalized it to 1,000 bp. For the estimation of intraspecific selection, we calculated the ratio of non-synonymous variants per non-synonymous site (pN) and synonymous variants per synonymous sites (pS). We then calculated the ratio of pN/pS for each gene and species [48].

## Authors' contributions

CK, SHY and JM worked on the gene discovery and primer design and all lab work. GFM was responsible for the plant material and DNA collections. GFM and SHY extracted DNA for one plant species. CK and GFM conducted the data analysis and wrote the first draft of the manuscript. WJF coordinated the study. All authors read and approved the final manuscript.

## Supplementary Material

Additional file 1 **Table of the gene copy number from A. thaliana, O. sativa, P. trichocarpa and V. vinifera**. Values estimated from BLAST searches and searches with the gene names. Values in brackets are unconfirmed genes or pseudogenes. The shading of the gene name indicates genes with a high copy number. Click here for file

Additional file 2 **pN/pS for each gene and species studied**. This file shows the values for non-synonymous SNPs per non-synonymous site divided by synonymous SNPs per synonymous sites (pN/pS) for each species Click here for file

Additional file 3 **Primer list for the set of 23 genes used in this study**. This file shows the primers used for each gene, the EST used for designing the primer and the length of each fragment amplified by the primer pair. Click here for file
