# Supplementary material for: Comparative SNP diversity among four Eucalyptus species for genes from secondary metabolite biosynthetic pathways
Source: BMC Genomics. 2009 Sep 24;10:452. doi: 10.1186/1471-2164-10-452 (PMC2760585; doi:10.1186/1471-2164-10-452)
Supplement: Additional file 1 — Table of the gene copy number from A. thaliana, O. sativa, P. trichocarpa and V. vinifera. Values estimated from BLAST searches and searches with the gene names. Values in brackets are unconfirmed genes or pseudogenes. The shading of the gene name indicates genes with a high copy number. [file 1471-2164-10-452-S1.doc]

Supplemental Table 2 The estimated gene copy number for four species with a sequenced genome is shown. We placed the genes into two categories, high copy number (shaded) and low copy number (not shaded).

|  | *A. thaliana* | *O. sativa* | *P. trichocarpa* | *V. vinifera* |
| --- | --- | --- | --- | --- |
| *dxs* | 3 | 3 | 6 | 4 |
| *dxr* | 1 | 1 | 2 | 1 |
| *hds* | 1 | 1 | 1(1) | (1) |
| *hdr* | 1 | 1(2) | 2 | 1 |
| *hmgs* | 1 | 3 | 2 |  |
| *mvk* | 1 | 1 | 1(1) |  |
| *pmd* | 2 | 3 | 2 |  |
| *ipp* | 2 | 2 | 1 | 1 |
| *ggpps* | 11 | 3 | 5 | 4* |
| *psy* | 1 | 3 | 6 |  |
| *gpps* | 1 | 2 | 2 | 4* |
| *fpps* | 2 | 5 | 5 | 4* |
| *smo* | 3(4) | 2 | 5 |  |
| *chs* | 1(3) | 29 | 8(3) | 4 |
| *chi* | 2(3) | 1(2) | 1 | 1 |
| *f3h* | 1 | 3 | 2 | 3 |
| *dfr* | 1(1) | 1 | 2 | 1 |
| *ans* | 1 | 0 | 2 | 1 |
| *lar* | 0 | 13 | 3 | 2 |
| *anr* | 1 | 0 | 2 | 1 |

* The copy number for the prenyl transferase family is described as four.
