# Supplementary material for: Comparative SNP diversity among four Eucalyptus species for genes from secondary metabolite biosynthetic pathways
Source: BMC Genomics. 2009 Sep 24;10:452. doi: 10.1186/1471-2164-10-452 (PMC2760585; doi:10.1186/1471-2164-10-452)
Supplement: Additional file 2 — pN/pS for each gene and species studied. This file shows the values for non-synonymous SNPs per non-synonymous site divided by synonymous SNPs per synonymous sites (pN/pS) for each species [file 1471-2164-10-452-S2.doc]

Supplemental Table 3: Ratio of non-synonymous variants per non-synonymous site to synonymous variants per synonymous sites for each gene and four species.

|  | *E. globulus* | *E. nitens* | *E.camaldulensis* | *E. loxophleba* | Average |
| --- | --- | --- | --- | --- | --- |
| *dxs1* | 0.05 | 0.06 | 0.10 | 0.11 | 0.08 |
| *dxs2* | 0.23 | 0.12 | 0.06 | 0.04 | 0.11 |
| *dxr* |  | 0.24 | 0.81 | 0.31 | 0.45 |
| *hds* | 0.80 | 0.39 | 0.35 | 0.25 | 0.45 |
| *hdr* | 0.21 | 0.28 | 0.22 | 0.25 | 0.24 |
| *hmgs* | 0.26 | 0.32 | 0.21 | 0.25 | 0.26 |
| *mvk* | 0.22 | 0.22 | 0.14 | 0.27 | 0.21 |
| *pmd* | 0.11 | 0.13 | 0.30 | 0.17 | 0.18 |
| *ipp* | 0.95 | 0.33 | 0.12 | 0.26 | 0.42 |
| *ggpps* | 0.20 | 0.20 | 0.15 | 0.06 | 0.15 |
| *psy1* | 0.31 | 0.26 | 0.36 | 0.17 | 0.28 |
| *psy2* | 0.73 | 0.38 | 0.36 | 0.22 | 0.42 |
| *psy3* | 0.25 | 0.61 | 0.36 | 0.49 | 0.43 |
| *gpps* | 0.16 | 0.82 | 0.33 | 0.13 | 0.36 |
| *fpps* | 0.15 | 0.91 | 0.24 | 0.35 | 0.41 |
| *smo* | 0.16 | 0.34 | 0.15 | 0.13 | 0.19 |
| *chs* | 0.56 | 0.85 | 0.59 | 0.75 | 0.69 |
| *chi* |  | 0.69 | 0.13 | 0.15 | 0.32 |
| *f3h* | 0.16 | 0.37 | 0.08 | 0.22 | 0.21 |
| *dfr* | 0.13 | 0.88 | 0.24 | 0.29 | 0.39 |
| *ans* | 0.37 | 0.26 | 0.22 | 0.21 | 0.26 |
| *lar* |  | 0.11 | 0.27 | 0.21 | 0.19 |
| *anr* |  | 0.32 | 0.19 | 0.08 | 0.19 |
